# job satisfaction and its impact on resilience: a cross-sectional study of Tunisian interns and residents in medicine

**DOI:** 10.1192/j.eurpsy.2022.1568

**Published:** 2022-09-01

**Authors:** F. Tabib, F. Guermazi, A. Zouari, S. Hentati, I. Baati, J. Masmoudi

**Affiliations:** 1 UHC Hedi Chaker, Department Of Psychiatry A, sfax, Tunisia; 2 UHC Hedi Chaker, Department Of Psychiatry A, Sfax, Tunisia

**Keywords:** interns, resilience, residents, job satisfaction

## Abstract

**Introduction:**

Preventing burnout and promoting resilience are important to the well-being of health care professionals and the quality of patient care. Indeed, it’s a promising way to mitigate the negative effects of stressors and allow professional growth.

**Objectives:**

study the association between job satisfaction and resilience in medical interns and residents.

**Methods:**

As part of a descriptive and analytical cross-sectional study, interns and medical residents completed an online self-questionnaire using ’Google Forms’. It collected socio-demographic data and assessed the level of job satisfaction using a 5-point Likert-type scale for each item. The Brief Resilience Scale (BRS) was used to assess the level of resilience.

**Results:**

The total number of participants was 56, of which 64.3% were medical residents.75% of the participants worked in a medical department and most had a number of shifts per month ≥4. The average years of practice was 2.27±1.23 years. Participants expressed dissatisfaction at work with salary (69.6%), task allocation and organization (66.1%), availability of resources (66.1%), comfort (57.1%), safety (53.6%) and supervision (50%). Referring to the BRS scale, higher resilience scores were objectified in male participants (p=0.002). The level of resilience decreased with the number of years of practice (p=0.039). Good satisfaction by management and recognition at work could enhance the level of resilience (p=0.029 and p=0.043 respectively).

**Conclusions:**

The results of our study suggest that dissatisfaction with work-related aspects may influence the level of resilience. These results deserve special attention to improve job satisfaction and preserve resilience.

**Disclosure:**

No significant relationships.

